# Docetaxel and Ramucirumab as Subsequent Treatment After First-Line Immunotherapy-Based Treatment for Metastatic Non-Small-Cell Lung Cancer: A Retrospective Study and Literature Review

**DOI:** 10.3390/curroncol32110612

**Published:** 2025-11-01

**Authors:** Sotiris Loizidis, Paris Vogazianos, Zoe Kordatou, Georgios Fotopoulos, George Orphanos, Flora Kyriakou, Haris Charalambous

**Affiliations:** 1 Medical Oncology Department, Bank of Cyprus Oncology Centre, Nicosia 2011, Cyprus; sotiris.loizides@bococ.org.cy (S.L.); zoe.kordatou@bococ.org.cy (Z.K.); georgios.fotopoulos@bococ.org.cy (G.F.); 2 Department of Social and Behavioral Sciences, School of Humanities, Social and Education Sciences, European University, Nicosia 2404, Cyprus; p.vogazianos@euc.ac.cy; 3 Medical Oncology Department, German Medical Institute, Limassol 4108, Cyprus; george.orphanos@goc.com.cy; 4 Medical Oncology Department, Mediterranean Hospital, Limassol 3117, Cyprus; flora.kyriakou@medihospital.com.cy

**Keywords:** immunotherapy, antiangiogenic agents, non-small-cell lung cancer, VEGF, docetaxel, ramucirumab

## Abstract

**Background**: A combination of docetaxel and ramucirumab represents a standard of care in second-line treatment for patients with advanced NSCLC. Evidence of the regimen’s efficacy is based on the results of the REVEL trial conducted in the pre-immunotherapy (immune checkpoint inhibitors–ICIs) era. Given the lack of randomized trials after the use of ICIs in front-line therapy, a question remains regarding the impact of the combination when disease progresses after ICI-based therapy. **Methods:** From 1 January 2018 to 31 December 2024, 55 patients from three oncology centers who had documented progression on ICI-based therapy subsequently received docetaxel/ramucirumab, and we reviewed their outcomes. **Results:** The studied group’s median progression-free survival (PFS) was 5.8 months, while the median overall survival (OS) was 11.1 months. The objective response rate (ORR) and disease control rate (DCR) were 42% and 76%, respectively. Patients who had received ICI-based therapy for ≥6 months had a numerically better median PFS and statistically significant OS compared to those who had experienced progression on ICI-based therapy in <6 months. Regarding adverse events (AEs), 92.7% of patients experienced Grade 1–2 AEs, whereas 54.5% experienced Grade ≥ 3 AEs. One death due to GI bleeding was also recorded. **Conclusion:** Docetaxel/ramucirumab is an acceptable regimen for patients progressing on first-line ICI-based therapies. Our results are in concordance with the REVEL study and other retrospective studies of this combination after ICIs.

## Simple Summary

The combination of docetaxel and ramucirumab is a standard second-line treatment for patients with advanced metastatic non-small-cell lung cancer (NSCLC); however, evidence supporting this regimen comes from the REVEL trial conducted before the introduction of immune checkpoint inhibitors (ICIs) in clinical practice. ICIs have now been approved as first-line therapy for metastatic NSCLC, raising the question of the efficacy of this combination after ICI failure. In our study, we report on the outcomes of 55 patients who received docetaxel/ramucirumab after progression on ICI-based therapy. Median progression-free survival (PFS) and overall survival (OS) were 5.8, and 11.1 months, respectively, whilst objective response rate and disease control rate were 42% and 76%, respectively. Patients who received ICI-based therapy lasting for ≥6 months had numerically better median PFS and a statistically significant increase in OS compared to those who were treated with ICI-based therapy for <6 months. Our results compare favorably with the REVEL study, providing further support for the use of this combination after ICIs.

## 1. Introduction

The introduction of immune checkpoint inhibitors (ICIs) has revolutionized the treatment of advanced non-small-cell lung cancer (NSCLC). Currently, ICIs are being used in combination with platinum-based therapies or as monotherapy for tumors with high expression of PD-L1 [1,2,3,4,5,6]. Despite the progress made with ICIs, most patients experience disease progression after the use of chemo-immunotherapy or single-agent ICI, requiring further treatment. An established second-line therapy is the combination of docetaxel and ramucirumab. Ramucirumab is a fully human IgG1 monoclonal antibody that selectively binds to the VEGFR-2 extracellular domain with high affinity. Evidence regarding the efficacy of this combination is based on the results of the randomized phase III clinical trial, REVEL [7]. The REVEL study was conducted before the introduction of ICIs in clinical practice for NSCLC and no randomized trials have been conducted in the ICI era to address this issue, i.e., to compare the addition of ramucirumab to docetaxel. Hence, it is unknown how well this combination performs after first-line chemo-immunotherapy or immunotherapy with ICI alone. Three small Japanese prospective trials reported on the use of docetaxel and ramucirumab after ICI-based first-line therapy [8,9,10]. Hence, evidence regarding the survival outcomes of the docetaxel/ramucirumab regimen after disease progression on ICI-based therapy has been primarily derived from retrospective studies and their meta-analyses [11,12,13,14,15,16,17,18,19,20,21,22,23,24].

The question regarding the impact of the combination has gained more interest, given the suggestion that previous treatment with ICI may potentiate the efficacy of docetaxel and ramucirumab. This relates to the understanding of the key role that VEGF/VEGFR plays as part of the resistance mechanism to ICIs, and that VEGF/VEGFR molecular signalling can act as an immunological modulator; when activated in lung cancer, it can render the tumor microenvironment (TME) immunosuppressive [25,26]. Currently, there is great interest in the potential impact of the combination of ICIs and anti-antiangiogenic agents in the first-line or second-line post-immunotherapy progression setting. There is evidence from the Harmoni-2 phase III clinical trial that ivonescimab, a novel bispecific antibody targeting both PD-1 and VEGF, can improve outcomes compared to the ICI often used, pembrolizumab [27], whilst similar studies with a combined approach of targeting both PD-1 and VEGF are ongoing. Furthermore, in the second-line setting, the phase II Lung-MAP S1800A trial, demonstrated that the combination of pembrolizumab with ramucirumab in patients progressing on ICI-based first-line therapy provides a survival advantage compared to standard second-line chemotherapy [28].

In our retrospective study, we primarily sought to investigate the effect of the docetaxel/ramucirumab combination in patients who had progressed after prior ICI-based therapies, as well as its toxicity and safety.

## 2. Materials and Methods

### 2.1. Patients

Using electronic databases from three oncology centers in Cyprus, we retrospectively retrieved the data of 55 patients who were treated with docetaxel and ramucirumab between 1 January 2018 and 31 December 2024, with a cut-off follow-up date of 3 April 2025. The latter date was selected to allow at least four months of follow-up data. The demographic data are listed in Table 1. In our clinical practice, the use of ramucirumab was limited to patients without recent hemoptysis or tumors close to or invading blood vessels, and patients with no uncontrolled hypertension.

For 50 of 55 patients, radiological assessment was available, according to Response Evaluation Criteria in Solid Tumors (RECIST) version 1.1. All had received ICI-based treatment and had documented disease progression. All patients received at least one cycle of docetaxel/ramucirumab immediately after ICI-based therapies.

### 2.2. Statistical Analysis

We calculated the median PFS and OS of the 55 patients who received docetaxel/ramucirumab. PFS was defined from the time of initiation of docetaxel/ramucirumab until the radiological progression of the disease. Seven patients were censored for PFS as they had not progressed at the time of the final analysis. OS was defined from the time of the initiation of treatment until death. Patients who were alive (n = 11) at the cut-off date (30 April 2025) or lost in follow-up (n = 2) were censored. Median PFS and OS were estimated by using Kaplan–Meier analysis. Comparisons between groups for a number of different variables, i.e., age, gender, histology, number of metastases, performance status, smoking history, and tumor proportion score (TPS), were estimated using the log-rank test. *p* < 0.05 was considered statistically significant. Statistical analyses were performed using SPSS version 29 and R version 4.4.1.

## 3. Results

### 3.1. Efficacy of Docetaxel/Ramucirumab

**Figure 1:**
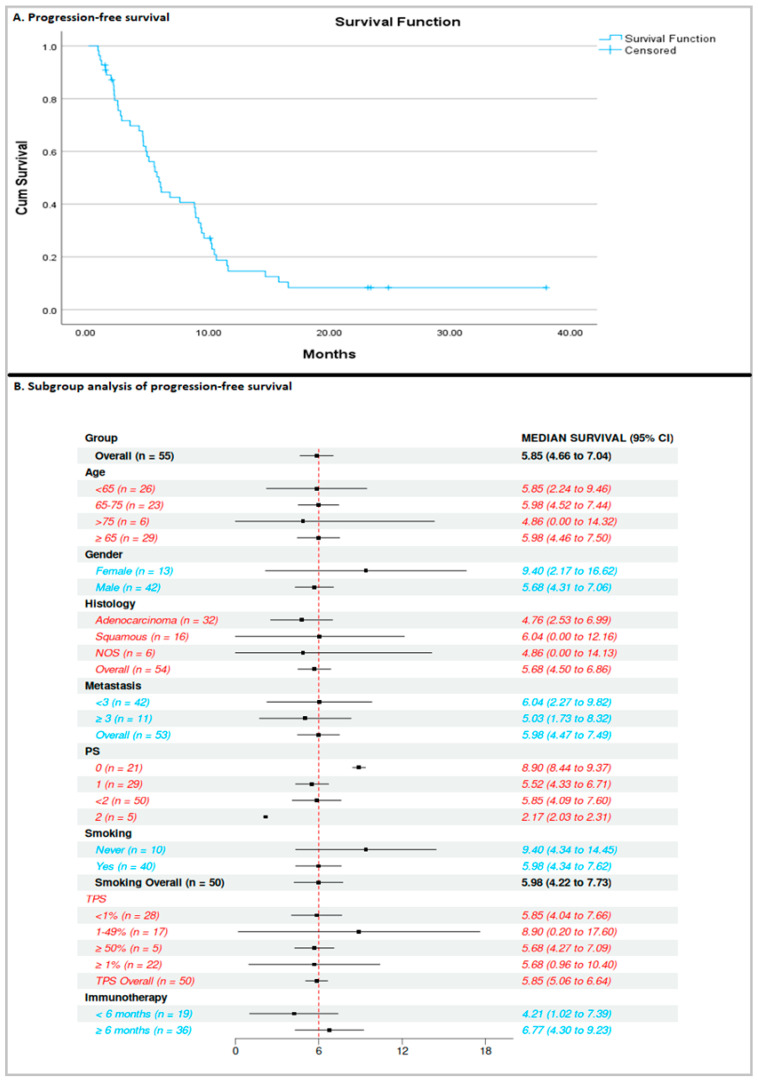
Progression-free survival of the population and subgroup analysis.

**Figure 2:**
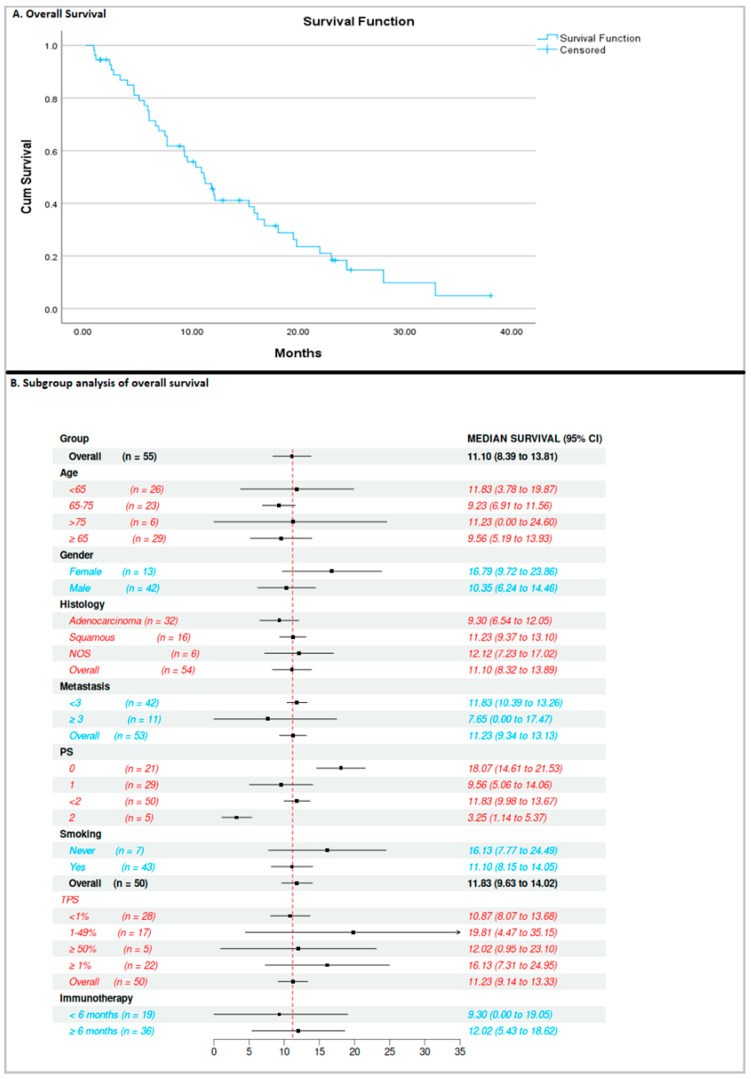
Overall survival of the population and subgroup analysis.

At the time of analysis, the median PFS (Figure 1) was 5.8 months (95% CI: 4.6–7) and was calculated based on 47 events (85.4% maturity), while the median OS (Figure 2) was 11.1 months (95% CI: 8.4–13.8), based on 42 events (76.3% maturity).

Subgroup analysis was undertaken according to age, gender, histology, number of metastases, performance status, smoking history, and TPS. The only significant finding was that patients with excellent performance status, PS = 0, had a better median PFS (*p* < 0.05) and OS than those with PS = 2 (8.9 months vs. 2.1 months and 18 months vs. 3.2 months, respectively). There was a trend toward better PFS in women, never smokers, and patients with a PDL-1 TPS score of 1–49%. A trend toward better OS was observed among women, never smokers, and patients with a PDL-1 TPS score of 1–49%, without reaching statistical significance.

### 3.2. Association Between Exposure Time to Previous ICI-Based Therapy and Efficacy of Docetaxel/Ramucirumab

We utilized the Society for Immunotherapy of Cancer (SITC) definitions of acquired resistance for patients progressing on ICI-based therapy after six months of treatment and primary resistance progressing within six months [29].

**Figure 3:**
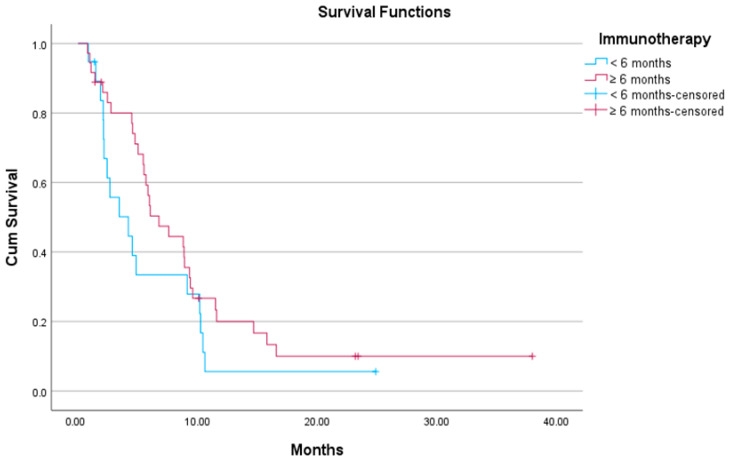
Kaplan–Meier plot showing median PFS of patients on docetaxel/ramucirumab who received ICI-based treatment lasting ≥6 months versus those who received ICI-based treatment for <6 months.

**Figure 4:**
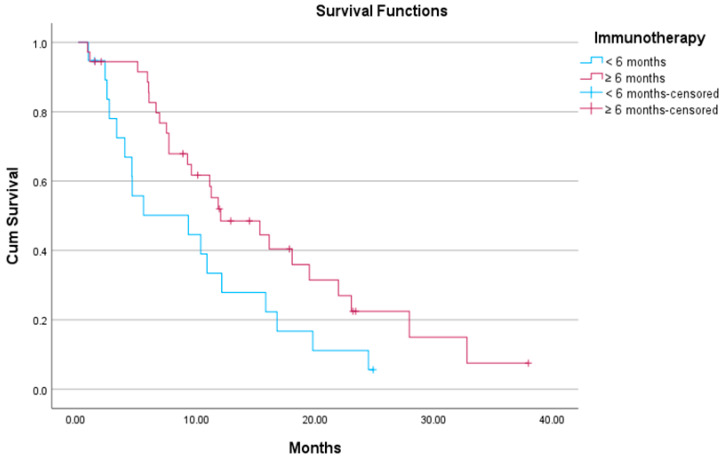
Kaplan–Meier plot showing median OS of patients on docetaxel/ramucirumab who had received ICI-based treatment for ≥6 months versus those who received ICI-based treatment for <6 months.

The median PFS of those patients who had received first-line ICI-based therapy for greater than 6 months was 6.7 months (95% CI: 4.3–9.2), while for those who had progressed within 6 months, the median PFS was 4.2 months (95% CI: 1–7.3) (Figure 3). The difference between groups was not statistically significant (*p*-value = 0.15). The median OS for the first group was 12 months (95% CI: 5.4–18.6), whereas for the latter, it was 9.3 months (95% CI: 0–19). The difference between the two groups was statistically significant (*p*-value = 0.04) (Figure 4).

### 3.3. Response Rates for Docetaxel/Ramucirumab

The ORR and DCR were calculated for 50 patients. For five patients, radiological assessments were not available and they were excluded from the calculations. ORR was defined as the proportion of patients who experienced partial response (PR) or complete response (CR) to therapy, while DCR was defined as the proportion of patients who achieved a response to treatment (PR or CR) and/or had stable disease (SD). In our population, none of the patients achieved CR, 21 experienced PR, 17 had SD, and 12 experienced disease progression (Table 2). ORR was 42% (95% CI: 28.3–55.7%), and DCR was 76% (95% CI: 64.1–87.8%).

### 3.4. Toxicity Profile

Table 3 lists the observed adverse events among the study population. Most patients experienced Grade 1 or 2 AEs (51/55; 92.7%). The most common AEs were malaise (50.9%), diarrhea (29.1%), and numbness (25.4%). A total of 54.5% of the patients experienced Grade 3–5 toxicity. There was one Grade 5 (death) due to GI bleeding, and four patients (7.3%) had Grade 3/4 diarrhea. Regarding ramucirumab, adverse events of special interest (AESIs) were experienced by thirteen patients (23.6%) and included ten (18.2%) hemorrhagic episodes, two (3.6%) thromboembolic events, one case (1.8%) of proteinuria, and one case (1.8%) of heart failure (Grade 2). Ten patients exhibited Grade 1–2 AEs. Regarding Grade 3 and above, there was one case of Grade 3 epistaxis, one case of Grade 3 hemoptysis, one case of Grade 3 proteinuria, and one case of Grade 5 GI bleeding. The AEs were graded according to the Common Terminology Criteria for Adverse Events (CTCAE) version 5.0.

## 4. Discussion

Data about the efficacy of combining docetaxel with ramucirumab after ICI-based therapy are mainly derived from retrospective studies (Table 4), except for three small Japanese phase II non-randomized prospective trials [8,9,10]. A recent systematic review by Garon et al. identified twelve studies in which docetaxel/ramucirumab demonstrated a median PFS and OS of 5.7 months and 11.2 months, respectively [11]. Moreover, in a recent multicenter retrospective study by Nakamura et al., which included 288 patients, the median PFS was 4.1 months, the median OS was 11.6 months, and the ORR was 28.8% [12]. These results strongly resemble our findings, with a median PFS of 5.8 months and a median OS of 11.1 months, which are, in fact, almost identical to those of the systematic review by Garon et al.

Our results and those of the systematic review by Garon et al. compare favorably with the results of the REVEL study and are numerically superior, especially regarding PFS, in which a median PFS of 4.5 months and median OS of 10.5 months were reported [7]. We postulate that using ICIs in first-line therapy may potentiate the effect of antiangiogenic agents in the second-line setting, probably due to the long-standing effects/half-life of ICIs, resulting in a synergistic effect with the addition of the anti-VEGF agents. This predominantly impacts PFS, as this interaction is only transient, given that the ICI has been stopped. Other studies have shown similar results; for instance, Kareff et al. performed a simulation comparison of their retrospectively collected data with REVEL’s results, showing a statistically significant difference in survival in favor of the docetaxel/ramucirumab regimen when used after ICI-based therapy [24]. Also of interest is a multicenter retrospective study by Tozuka et al., which showed a statistically significant difference in terms of PFS in those who received docetaxel/ramucirumab and had prior exposure to ICIs compared to those who did not [17], again suggesting a potential synergistic effect of previous ICI exposure and the subsequent use of ramucirumab.

Similar to the above data for docetaxel and ramucirumab, the combination of another oral antiangiogenic drug, nintedanib, with docetaxel was evaluated in the prospective VARGADO trial (Cohort C), also suggesting numerically better results post-ICI compared to the original LUME LUNG 1 trial. The VARGADO trial recruited 137 patients who had progressed on first-line ICI-based therapy, showing an ORR of 37.5% and median PFS of 4.8 months, while the median OS was immature at the time of analysis [30].

In our survey, patients who had received more than 6 months of first-line ICI-based therapy had numerically better PFS and statistically significant OS with second-line docetaxel/ramucirumab treatment than those whose ICI-based therapy lasted less than 6 months. The lack of statistical significance for PFS could be attributed to the relatively small sample size, especially taking into account the difference in median PFS in the ≥6 months vs. the <6 months groups, which was 6.7 months vs. 4.2 months (2.5 months absolute difference). A similar trend was observed in OS, with median values of 12 months vs. 9.3 months (2.7 months absolute difference). This may reflect differences between primary and acquired ICI resistance. Similar data from by Ishida et al. demonstrated that patients who received long-term treatment (≥8.8 months) with ICIs had better PFS on a second-line regimen than those with shorter exposure to ICIs in the first-line setting (median PFS: not reached vs. 4.1 months, *p*-value = 0.003) [21].

Regarding older patients, in the REVEL study, there was a suggestion from a subset analysis of a lack of benefit from the combination of docetaxel and ramucirumab in patients over 70 years old, with the forest plot for OS showing an HR of 1.07, and for PFS, an HR of 0.94 [7]. This is, however, not confirmed in our study, where the outcomes of the subgroup of over-65-year-old patients were not inferior compared to those of their younger counterparts and this was consistent with other retrospective studies specifically looking at elderly patients. In our study, 60% of the participants were ≥65 years old, with the median PFS and OS for this subgroup being 5.9 and 9.5 months, respectively. Excellent results using the combination for elderly patients (≥70 years old) were reported by Onoi K. et al., demonstrating a median time to treatment failure of 6.3 months and a median OS of 15.9 months [31]. Furthermore, another retrospective study by Matsumoto et al. showed a median OS of 14.8 months and no difference in OS for patients ≥65 years compared to younger patients, while it was also demonstrated that a reduction in docetaxel dose could improve tolerability [32]. Nevertheless, it should be noted that these two studies included a mixed population of patients, both previously ICI-exposed and non-ICI-exposed. For this specific subset of elderly patients who received prior ICI-based therapy, Nakamura et al. reported in their large multicenter retrospective study from Japan that there were no statistically significant differences between individuals aged ≥75 years and their younger counterparts [12]. Finally, the multicenter German study by Brueckl et al. demonstrated that patients ≥65 years (36/77, 46.8%) had no inferior outcomes compared to those <65 years, reporting a median PFS of 3.5 months and 4.5 months (*p*-value = 0.101), respectively [19].

It is worth considering the mechanism of the potential synergism between ICIs and ramucirumab. This is based on evidence suggesting that angiogenesis exerts immunosuppressive effects and is implicated in resistance mechanisms to ICIs [26,33]. It has been shown that VEGF-A impairs the antigen-presenting abilities of dendritic cells and their maturation and mobilizes myeloid-derived suppressor cells (MDSC) from bone marrow to the TME [26]. It was also noted that in lung cancer, VEGF-A inhibits CD8+ T-cell activation, leading them to exhaustion [34]. Moreover, VEGF-A and VEGFR-2 are expressed by subpopulations within TME with immunosuppressive abilities, such as M2 macrophages and Foxp3+ T-regulatory cells [35]. Finally, in preclinical murine models, dual blockade of VEGFR-2 and PD-L1 has shown potent tumor inhibition and the development of immunological memory [36].

Based on the above evidence, it has been proposed that drugs targeting angiogenesis can normalize the abnormal tumor vasculature, which leads to an increase in the infiltration of immune effector cells into tumors and change the intrinsically immunosuppressive TME to an immune-supportive one, leading to increased efficacy of ICIs when used in combination [28,37]. In fact, the combination of ICIs and anti-angiogenic drugs has been tested in a variety of different cancer types. Currently, such combinations are the standard of care in the first- and second-line treatment of renal cell cancer, endometrial cancer, and hepatocellular carcinoma and are also used in some patients with NSCLC [38,39,40,41].

Regarding the combination of ramucirumab and ICIs, there is further support for their synergism in the clinical setting from the randomized phase II trial Lung-MAP S1800A, which demonstrated a survival benefit in terms of overall survival (14.5 months vs. 11.6 months, HR: 0.69, 95%CI: 0.51–0.92) for the combination of ramucirumab with the anti-PD-1 agent, pembrolizumab, compared to standard second-line therapy (predominantly docetaxel-based), for patients who had experienced progression on chemoimmunotherapy [28].

Ramucirumab is an FDA and EMA-approved anti-angiogenic antibody for NSCLC and gastric cancer, which inhibits VEGFR-2. Pre-clinical studies were undertaken in mice tumor models, where VEGFR-2 and PD-L1 monotherapies induced both unique and overlapping patterns of immune gene expression, and combination therapy resulted in an enhanced immune activation signature explaining the enhanced clinical activity observed in the combined blockade of VEGFR-2 and PD-1-axis pathways [42]. Given the above clinical and pre-clinical data about the potential synergism between ramucirumab and immune checkpoint inhibitors, especially pembrolizumab, and given the known efficacy of docetaxel in the second-line setting, Badi El Osta and colleagues are currently running a phase II trial examining the triplet combination of docetaxel, pembrolizumab and ramucirumab as second-line therapy in patients with metastatic NSCLC who have progressed after chemotherapy and immunotherapy [43].

Our study shows that patients with excellent performance status derived greater benefits from the combination of docetaxel and ramucirumab. Of note, in the S1800A study, patients with both squamous histology and excellent performance status (PS = 0) derived more benefit from the combination of pembrolizumab and ramucirumab [28]. The improved outcomes for patients with excellent performance status may relate to their ability to receive more chemotherapy and ramucirumab. However, the reason for the improved outcomes in patients with squamous histology is currently unclear.

Regarding the toxicity profile, the most common AEs in our study were malaise (50.9%), diarrhea (29.1%), and numbness (25.4%). A total of 54.5% of patients experienced Grade ≥3 toxicity. In the prospective SCORPION study on docetaxel in combination with ramucirumab after ICI-based therapy, where prospective toxicity data collection was undertaken, Grade ≥3 toxicity was noted in 58% of the participants (19/33), whilst the most common AE was malaise, as in our study [8]. In the other prospective study, Grade ≥3 toxicity was noted in 41.9% of the study population (18/43), while the most commonly reported AEs were neutropenia (30.2%), stomatitis (11.6%) and thrombocytopenia (11.6%) [9]. A Japanese post-marketing safety study included 401 patients who had received the combination, demonstrating AEs of any grade in 81.2%, while Grade ≥3 AEs were reported in 43.7%. The most common AEs were malaise (14.3%), decreased appetite (13%), and neutropenia (11.6%) [10]. Furthermore, our toxicity data appear to be better than those in the REVEL study, where adverse events of any grade were seen in 98% of patients in the ramucirumab/docetaxel arm, whilst the most common Grade 3 or worse AEs were neutropenia (49%), febrile neutropenia (16%), fatigue (14%), and hypertension (6%). The low rate of febrile neutropenia (1.8%) in our study is attributed to the primary prophylaxis of patients with G-CSF when using docetaxel, a practice supported by the literature [44]. Furthermore, in the REVEL study, 5% of patient deaths were from AEs and Grade ≥3 pulmonary hemorrhages in 1% of patients, whilst in our study, Grade ≥3 toxicity was seen in one patient with epistaxis (1.8%), one case of hemoptysis (1.8%), one case of proteinuria (1.8%), with one death due to GI bleeding (1.8%).

Our study’s limitations include its retrospective nature and the small sample size. We believe the sample size and retrospective nature of our study have not influenced the results, as the sample size is comparable to other similar studies published in this field, and the majority of the studies published are retrospective. Furthermore, the results are in line with all the other publications in this field, as can been seen in Table 4. The advantages of our study include treatment of patients by five site-specialized thoracic oncologists and that it is only the third study from Europe, with Caucasian, non-Asian patients (the other two studies come from a German collaborative group by Brueckl et al.). Although there are two studies from the same cancer center in Miami, by Dawar and Kareff et al., the rest of the studies come from Asia; therefore, our study provides more data about the applicability of this combination in Caucasian patients.

Notably, studies in Asia have a high proportion of patients, in fact, the majority, with actionable genomic alterations (AGAs), especially patients with EGFR mutations. In contrast, studies with Caucasian patients have a much lower proportion of patients with AGAs, for instance, in our series, there were only five patients with AGAs—less than 10%. It is important to make the point that patients with AGAs should receive docetaxel/ramicuramab after both targeted therapy and the use of chemo-immunotherapy. This was also practiced in our series; docetaxel and ramuricumab should only be used once all targeted therapy options have been exhausted and after platinum doublet immunotherapy combinations.

Our study confirms the findings of other research regarding the efficacy of docetaxel and ramucirumab, which is at least as effective as that reported in the REVEL study, in patients with advanced/metastatic NSCLC who progressed after first-line ICI-based therapy. Therefore, this study supports the use of docetaxel in combination with ramucirumab as second-line therapy after progression to ICI-based therapy.

## Figures and Tables

**Table 1:** Patient demographics.

Patient Characteristics	Number of Patients = 55 (%)
**Age, median** **(range)**	64.8 (36–80)
**<65**	22 (40)
**65–75**	27 (49.1)
**>75**	6 (10.9)
**Gender**	
**Male**	42 (76.4)
**Female**	13 (23.6)
**Performance Status**	
**0**	21 (38.2)
**1**	29 (52.7)
**2**	5 (9.1)
**Histology**	
**Adenocarcinoma**	32 (58.2)
**Squamous**	16 (29.1)
**NOS**	5 (9.1)
**Other**	2 (3.6)
**Smoking history**	
**Pack/years (median)**	53.7
**Active/Ex-smokers**	43 (78.2)
**Never smokers**	7 (12.7)
**N/A**	5 (9.1)
**TPS**	
**<1%**	28 (50.9)
**1–49%**	17 (30.9)
**>50%**	5 (9.1)
**N/A**	5 (9.1)
**Molecular profiling**	
**No mutations**	29 (52.7)
***EGFR***	3 (5.4)
***ALK***	-
***KRAS***	7 (12.7)
Other *	13 (23.6)
**N/A**	7 (12.7)
**Sites of metastases**	
**≥3 sites**	11 (20)
**<3 sites**	42 (76.4)
**Relapsed Stage III**	2 (3.6)
**IO drug**	
**Pembrolizumab**	48 (87.4)
**Nivolumab**	2 (3.6)
**Atezolizumab**	2 (3.6)
**Nivolumab/Ipilimumab**	3 (5.4)
**Prior immunotherapy type of regimen**	
**Monotherapy**	7 (12.7)
**Chemo-IO**	48 (87.3)
**Docetaxel/Ramucirumab line of therapy**	
**Second**	46 (83.6)
**Third or beyond**	9 (16.4)

* Other: 1 *AKT1* amplification; 1 *CDK6* amplification; 4 *EGFR* amplification; 2 *FGFR1* amplification; 1 *MET*ex14; 1 *MYC* amplification; 1 *NRAS* mutation; 1 *PIK3CA* mutation; 1 *RET* rearrangement. Abbreviations: IO, immunotherapy; NOS, not otherwise specified; TPS, tumor proportional score.

**Table 2:** Response rates in the studied population.

Overall Response	N = 50 (%)
**CR**	-
**PR**	21 (42)
**SD**	17 (34)
**PD**	12 (24)
**ORR**	21/50 (42)
**DCR**	38/50 (76)

Abbreviations: CR, complete response; DCR, disease control rate; PD, progressive disease; PR, partial response; ORR, objective response rate; SD, stable disease.

**Table 3:** Toxicity profile.

Adverse Events	Grade 1–2, N (%)	Grade ≥ 3, N (%)
**Any**	51 (92.7)	30 (54.5)
**Malaise**	22 (40)	6 (10.9)
**Numbness**	13 (23.6)	1 (1.8)
**Diarrhea**	12 (21.8)	4 (7.3)
**Nausea**	6 (10.9)	1 (1.8)
**Epiphora**	6 (10.9)	-
**Constipation**	5 (9.1)	-
**Anorexia**	5 (9.1)	-
**Cough**	5 (9.1)	-
**Hypertension**	5 (9.1)	1 (1.8)
**Anemia**	4 (7.3)	5 (9.1)
**Mucositis**	4 (7.3)	-
**Dysgeusia**	3 (5.4)	-
**Hoarseness**	3 (5.4)	-
**Alopecia**	2 (3.6)	-
**Hypothyroidism**	2 (3.6)	-
**Pruritus**	1 (1.8)	-
**Vomiting**	1 (1.8)	-
**Skin rah**	1 (1.8)	3 (5.4)
**Neutropenia**	1 (1.8)	3 (5.4)
**Thrombocytopenia**	1 (1.8)	1 (1.8)
**Febrile Neutropenia**	-	1 (1.8)
**AESIs of Ramucirumab**		
**Heart failure**	1 (1.8)	-
**Bleeding or hemorrhage**		
**Hemoptysis**	2 (3.6)	1 (1.8)
**GI bleeding**	2 (3.6)	1 (1.8)
**Epistaxis**	2 (3.6)	1 (1.8)
**Menorrhagia**	1 (1.8)	-
**Thromboembolic events**		
**PE**	1 (1.8)	-
**DVT**	1 (1.8)	-
**Proteinuria**	-	1 (1.8)

Abbreviations: AESIs, adverse events of special interest; DVT, deep vein thrombosis; PE, pulmonary embolism.

**Table 4:** Studies (chronologically ordered) evaluating the benefit of docetaxel/ramucirumab after the failure of first-line ICI-based therapy.

Study’s First Author, Year of Publication [Reference]	Study Type	Number of Patients	PFS (Months)	OS (Months)	RR (%)
**Harada, 2019** [12]	Retrospective, single center	18	5.7	13.8	38.9
**Shiono, 2019** [13]	Retrospective, single center	20	5.6	11.3	60
**Yoshimura, 2019** [14]	Retrospective, multicenter	40	5.7	11.9	NA
**Kato, 2020** [15]	Retrospective, multicenter	62	5.7	17.5	20.9
**Tozuka, 2020** [16]	Retrospective, single center	21	5.9	19.8	42.6
**Brueckl, 2020** [18]	Retrospective, multicenter	67	6.8	11	36
**Brueckl, 2021** [19]	Retrospective, multicenter	77	3.9	7.5	32.5
**Dawar, 2021** [19]	Retrospective, single center	35	6.6	20.9	NA
**Ishida, 2021** [20]	Retrospective, multicenter	18	5.8	10.7	55.6
**Nishimura, 2022** [22]	Retrospective, single center	17	2.4	7.2	57.1
**Tanizaki, 2023** [23]	Retrospective, multi-center	94	5.8	13.4	31
**Chen, 2022** [10]	Prospective, multicenter	154	NA	58.7% *	NA
**Kareff, 2023** [24]	Retrospective, single center	35	6.6	20.9	NA
**Matsuzawa, 2023** [8]	Phase II, single arm	32	6.5	17.5	34.4
**Nakamura, 2023** [12]	Retrospective, multicenter	288	4.1	11.6	28.8
**Katayama, 2024** [9]	Prospective, multicenter	44	6.3	22.6	36.4

Abbreviations: NA, not available; OS, overall survival; PFS, progression-free survival; RR, response rate. * The authors reported outcomes only in terms of the 12-month survival rate.

## Data Availability

Data supporting the reported results can be found at: https://drive.google.com/file/d/1869L7dIW1YxraDJo5mpjiJNtnyLJekzm/view?usp=drive_link. (accessed on 1 October 2025).
